# Cross-sectional associations between occupational factors and musculoskeletal pain in women teachers, nurses and sonographers

**DOI:** 10.1186/s12891-016-0883-4

**Published:** 2016-01-18

**Authors:** Inger Arvidsson, Jenny Gremark Simonsen, Camilla Dahlqvist, Anna Axmon, Björn Karlson, Jonas Björk, Catarina Nordander

**Affiliations:** Division of Occupational and Environmental Medicine, Lund University, SE-221 85 Lund, Sweden

**Keywords:** Musculoskeletal disorders, Mechanical exposure indices, Technical measurements, Copenhagen psychosocial questionnaire, Multivariable model

## Abstract

**Background:**

It is usually assumed that musculoskeletal pain is associated with both the physical workload and the psychosocial work environment, as well as with personal and lifestyle factors. This study aims to ascertain the prevalence of musculoskeletal pain in women with varying or different occupational exposures, and to explore the associations between musculoskeletal pain and the occupational and personal factors.

**Methods:**

A questionnaire on physical, psychosocial and individual factors was answered by 1591 women in five occupational groups with contrasting occupational exposures (teachers, anaesthetic, theatre, and assistant nurses, and sonographers). The outcome measure was musculoskeletal pain (in a new model based on frequency and intensity of complaints the preceding year) from the neck, shoulders, hands, lower back and feet.

**Results:**

Neck pain was equally frequent among teachers, assistant nurses and sonographers, and less frequent in anaesthetic and theatre nurses. The sonographers experienced the highest prevalence of shoulder pain, while the assistant nurses were the most affected in the wrists and hands, lower back, and feet.

The teachers reported the highest scores in most of the psychosocial dimensions. The theatre nurses scored highest in strenuous work postures and movements (mechanical exposure index, MEI), and the assistant nurses in physical activity and lifting (physical exposure index, PHYI). Multivariable models in the total population showed that both the physical workload and the psychosocial work environment were associated with pain in all body regions, though different factors affected different regions. Pain in the neck, shoulders, hands and lower back was strongly associated with a high MEI and high job demands, while pain in the feet was associated with a high PHYI and a high BMI. A young age was associated with pain in the neck, and an older age was associated with pain in the hands and feet. Lack of time for personal recovery was associated with pain in the shoulders and lower back.

**Conclusions:**

The occupational groups were affected differently and need different protective measures. For the teachers, the psychosocial work environment should be improved. The surgical staff and sonographers require measures to mitigate lifting and constrained postures.

**Electronic supplementary material:**

The online version of this article (doi:10.1186/s12891-016-0883-4) contains supplementary material, which is available to authorized users.

## Background

Musculoskeletal disorders are a major public health problem, and a large proportion of these disorders are assumed to be associated with adverse work conditions. Increased risk has been demonstrated in occupations with highly repetitive work tasks, forceful exertions, awkward postures, and heavy lifting [1–4], as well as in demanding psychosocial work environments [5, 6]. Furthermore, gender, and personal and lifestyle factors, have implications for the incidence of symptoms [7, 8]. Various risk factors for musculoskeletal disorders among groups with a high physical workload are therefore recognised, but it is still unclear which factors are associated with disorders in common occupations – involving many individuals – with low or medium-level physical workloads. Health professionals suffer a relatively high prevalence of disorders in the back, neck and shoulders [9, 10]. In spite of including a diversity of tasks, their work includes several known physical risk factors. For example, theatre nurses’ work is characterized by prolonged twisted and static postures, which are associated with assisting with surgery and instrumentation. This applies also to sonographers performing ultrasound examinations [11, 12]. Assistant nurses in surgical wards perform heavy handling of patients, as well as of equipment. Anaesthetic nurses experience both static postures, for example, when anaesthetising and monitoring the patient, and heavy handling, as when lifting patients. In a pilot study of these professions, we found that the work was perceived as very stimulating despite the physical exertion. Schoolteachers, on the other hand, are considered to experience a high level of psychosocial stress [13], while physically their work is varied and relatively light. Together, these five professional groups therefore provide a high degree of contrast in their physical and psychosocial risk factors, which makes them interesting to study concerning preventable risk factors for musculoskeletal disorders.

### Aim

The aim of the present study, which comprises the baseline for a planned prospective cohort study, was to ascertain the prevalence of musculoskeletal pain in the neck, shoulder, hands, lower back and feet in women with varying or different occupational exposures; and to explore the associations between musculoskeletal pain and the women’s physical workload, psychosocial work environment and personal factors.

## Methods

### Study design

A questionnaire was directed to women from five occupational groups: teachers (Te), anaesthetic nurses (AnN), theatre nurses (TN), assistant nurses (AsN), and sonographers (Sg). A subgroup of 485 women (93–103 in each occupation) was also given a clinical examination of the neck and upper limbs. Postures, movements, and muscular load in the upper body were quantified by direct technical measurements of representative subgroups (12–13 subjects) of each occupation. In a multivariate model, musculoskeletal pain from five body regions was analysed in relation to 18 items of self-assessed work-related exposures and personal factors. Written informed consent for participation was obtained from all participants, in every part of the study. The regional ethics committee at Lund University approved the study (March 10 2010; reference no. 2010/19).

### Study populations

An invitation to take part in the study was extended to 64 state-run schools from seven counties in southern Sweden, all 23 surgical departments coming under the remit of County councils in southern Sweden, and all 45 departments at hospitals in Sweden where biomedical scientists perform sonography. Of these, 49 public schools, 22 surgical departments and all 45 sonography departments agreed to participate. In total, the questionnaire was sent to 2078 women at 116 different work sites during the period October 2008–December 2012. In order to avoid the results being affected by major changes in society during the lifetime of the study, questionnaires were sent to a subset each of the various employee categories alternately. At each work site, all women employed as Te, AnN, TN, AsN and Sg, were included if they had worked full time, or part-time at least 50 %, for at least 3 months. Among the teachers, only those who were teaching theoretical subjects in compulsory school years 4–9 were included. The sonographers were all professional biomedical scientists. Of the 2078 women, 1591 (77 %) submitted responses, divided between 375 Te (69 %), 297 AnN, 305 TN, 323 AsN (surgical staff in aggregate, 77 %), and 291 Sg (86 %).

Among the participants, women from 23 randomly-chosen work sites (four schools, four surgery departments, and 15 sonography departments) were invited to participate in an in-depth examination, with an interview about their working conditions, and a clinical examination of the neck and upper limbs. Of 505 women invited, 485 participated (96 Te, 94 AnN, 99 TN, 93 AsN and 103 Sg; participation rates among the groups were 93–99 %). Personal characteristics are given together with exposure factors in Table 1.

**Table 1:** Self-reported personal factors and exposures

		Total study population		Te	AnN	TN	AsN	Sg
	Scale	N		(*N* = 375)	(*N* = 297)	(*N* = 305)	(*N* = 323)	(*N* = 291)
Personal and lifestyle factors								
Age, years; mean (SD)		1591	47 (11)	47 (11)	47 (10)	47 (10)	50 (9)	44 (13)
BMI, points; mean (SD)		1555	24 (3.8)	24 (3.8)	24 (3.3)	24 (4.0)	25 (4.1)	24 (3.5)
Number of children at home; mean (SD)		1575	1.0 (1.1)	1.1 (1.1)	1.1 (1.1)	1.0 (1.0)	1.0 (1.1)	0.8 (1.0)
Personal recovery time (%)		1555						
≥ 3 h/day			25	24	18	26	28	31
1–2 h /day			53	53	59	51	52	48
< 1 h/day			22	23	23	23	20	21
Domestic work (%)		1564						
0–10 h/week			36	32	32	35	38	43
11–20 h/week			41	48	36	38	45	36
≥ 21 h/week			23	20	33	27	17	21
Physical exercise (%)		1570						
Twice a week or more			73	70	81	71	71	72
Once a week			14	14	13	14	12	17
Occasionally or never			13	15	6	15	17	11
Smoking habits (%)		1520						
Have never smoked			62	67	65	57	46	76
Former smoker			29	23	29	34	39	20
Smoking, but not daily			4	5	4	3	5	2
Daily smoker			5	4	2	6	10	2
Working hours/week (%)		1581						
Part-time <30 h/week			9	10	11	9	8	6
Fulltime ≥ 30 h/week			91	90	89	91	92	94
Physical workload								
MEI; mean (SD)	11–33	1484	19 (3.9)	16 (3.4)	19 (3.5)	21 (3.4)	20 (3.7)	18 (3.1)
PHYI; mean (SD)	7–21	1491	12 (3.2)	10 (1.7)	13 (3.0)	13 (2.7)	15 (2.9)	10 (2.1)
Satisfied with computer								
workstation arrangements (%)		1499						
Satisfied			42	28	36	39	47	64
Neutral			32	34	30	42	32	24
Dissatisfied			26	38	34	20	21	12
Psychosocial factors; mean (SD)								
Job demands	1–4	1548	2.8 (0.4)	2.9 (0.4)	2.9 (0.4)	2.8 (0.4)	2.8 (0.4)	2.5 (0.4)
Job control	1–4	1549	2.9 (0.4)	3.2 (0.3)	3.0 (0.3)	2.9 (0.4)	2.7 (0.3)	2.8 (0.3)
Job support	1–4	1552	2.9 (0.4)	2.8 (0.4)	2.8 (0.4)	2.9 (0.4)	2.9 (0.4)	2.9 (0.4)
Emotional demands	0–4	1530	2.2 (0.8)	2.9 (0.7)	2.2 (0.6)	2.0 (0.6)	1.9 (0.8)	1.8 (0.6)
Demands of hiding emotions	0–4	1528	1.6 (0.8)	1.7 (0.8)	1.6 (0.7)	1.6 (0.7)	1.4 (0.9)	1.7 (0.7)
Sensory demands	0–4	1530	2.9 (0.7)	2.4 (0.6)	3.1 (0.5)	3.3 (0.5)	2.8 (0.6)	3.2 (0.5)
Leadership	0–4	1528	2.0 (0.7)	2.0 (0.8)	1.9 (0.7)	2.0 (0.7)	2.1 (0.7)	2.2 (0.7)
Self-efficacy	1–5	1522	1.9 (0.5)	1.9 (0.5)	1.8 (0.4)	1.9 (0.5)	1.9 (0.5)	1.9 (0.4)

Personal and lifestyle factors (age, BMI, number of children, time for recovery, domestic work, physical exercise and smoking habits), working hours/week, and self-reported physical workload (mechanical and physical exposure indices, and computer workstation arrangements) and psychosocial work environment (job demands, job control, job support, emotional demands, demands of hiding emotions, sensory demands, leadership and self-efficacy) in the total study population, and separately among teachers (Te), anaesthetic nurses (AnN), theatre nurses (TN), assistant nurses (AsN) and sonographers (Sg); all women

### Work tasks

Teachers (Te): This work studied those teaching theoretical subjects in years 4–9 of the state school system, which educates children aged 10–15. Besides the teaching tasks, the Te act as mentors for pupils, are in contact with parents, and perform computerized administrative work.

Anaesthetic nurses (AnN): The AnN prepare the patient for surgery by inserting needles and intravenous drips, preparing drugs and equipment, and anesthetizing the patient by intubation. The AnN also help other personnel in turning, lifting and transferring the patient. During surgery, the AnN sit by the patient’s head, beside the operating table. They check instruments monitoring such things as oxygen saturation, blood pressure, and fluid balance, to ensure that the patient’s general health is maintained during surgery. They also provide pain relief. After the operation, the AnN wakes the patient and transfers them to post-op. They also move operating tables, beds and other equipment within the surgical ward.

Theatre nurses (TN): The TN prepare for surgery by laying out instruments, and help to place the patient on the operating table. TN are responsible for sterility in the operating theatre; they perform the sterile washing of the patient, and dress them in sterile clothing. During surgery, the TN stand beside the surgeon, assisting with instrumentation. The TN may, for example, hold a surgical retractor to hold the incision open. After the operation, the TN dress the surgical wound, and sometimes apply plaster casts.

Assistant nurses (AsN): The AsN prepare for the operation by laying out surgical instruments, and open a variety of packages with gloves and other materials. The AsN also move trolley carts with X-ray and other machinery into the operating room, sometimes offloading them; they connect any needed machines, and adjust the operating lights. Then, the AsN transport the patient into the operating theatre and move them from the bed to the operating table. The AsN adjust the patient’s position and place their extremities into footrests and armrests on the operating table. In addition, the AsN pre-washes (non-sterile) the patient, shaves them, and inserts any needed catheters. During surgery the AsN assist the other personnel in the operating theatre. After surgery, the AsN close down the operating theatre by cleaning, disposing of waste, washing receptacles, and re-stocking with new materials. Compared to the other four groups, the AsN have a lower level of education.

Sonographers (Sg): The main work tasks among the Sg include ultrasound examinations of the heart (echocardiography), the blood vessels, or other organs. The Sg manage an ultrasound machine, consisting of a screen, a keyboard, and a transducer attached with a cable. While the patient is lying on an examination table, the Sg sit or stand at their side, holding the transducer in one hand and operating the keyboard with the other. Simultaneously, they observe the screen. After the examination, the Sg analyse the results, either at the ultrasound machine or at a computer workstation, and write a report, including review and analysis of the images. Sometimes the Sg perform “bedside examinations” – examinations carried out in the ward with the patient lying in a hospital bed.

### Questionnaire

The questionnaire included questions about the physical workload, psychosocial working conditions, personal and lifestyle factors, and musculoskeletal pain from five different body regions.

Physical workload: The mechanical exposure index (MEI [14]) included 11 items of work postures and movements. The physical exposure index (PHYI [14]) included seven items concerned with physical activity and lifting. Each item was answered on a three-point scale; 1:”hardly nothing/not at all”, 2:”somewhat” or 3:”a great deal”. For each scale, the sum of the points (MEI 11–33 possible; PHYI 7–21 possible) was calculated for each individual. The ergonomic conditions during computer work were assessed by the question “Are you satisfied with the computer work-station arrangements?” with the options 1: “very satisfied (can work comfortably)” or “rather satisfied”, 2: “neither satisfied nor dissatisfied”, 3: “rather dissatisfied” or “very dissatisfied (uncomfortable/strenuous work)”.

Psychosocial work environment: The psychosocial exposure in terms of job demands, job control and job support was measured with a Swedish version of the Job Content Questionnaire (JCQ) [15, 16]. Job demands concerned nine items of e.g. working pace, hard work, excessive demands, time pressure, conflicting demands, and stressful work. Job control concerned nine items of decision latitude (e.g. influence at work, freedom to decide how work should be done) and skill discretion (e.g. development opportunities, skill and creativity). Job support included eight items concerning support from management and co-workers. Responses concerning each item used a four-point scale, indicating the level of agreement with various statements about conditions at work. The mean value in each dimension was calculated for each individual. Higher numbers indicated higher demands, better control, and better support.

A subset of the Copenhagen Psychosocial Questionnaire [17] was used to measure dimensions defined as emotional demands (three items concerning e.g. emotionally difficult situations and emotional affection by the work), demands on hiding emotions (two items), sensory demands (five items concerning e.g. eye sight, attention, control of body movements and precision), and leadership (eight items concerning planning of work, conflict solving, communication and concern for staff). All questions were answered on a 5-point-scale and the mean value in each dimension was calculated for each individual.

Self-efficacy was assessed with three items [18]: The respondents were asked to judge whether three statements matched themselves: “You can deal with most unexpected events”, “You can solve most problems if you really want to” and “Irrespective of what is going on in your life, you feel that you can handle it”: All items had five response categories: always, often, sometimes, seldom, and never/ hardly ever. The mean score (range 1–5) was used as a continuous predictor. Higher scores indicated greater self-efficacy.

Personal and lifestyle factors: the participants were asked about their age, height and weight (body mass index, BMI, calculated as (weight in kg)/(height in meters)^2^) and the number of children they had at home. They were also asked “How much of your leisure time do you normally use for personal recovery?” (1: ≥3 h/day; 2: 1–2 h/day; and 3: <1 h/day) and “How many hours a week, do you normally work at home doing cleaning, gardening, cooking, etc.?” (domestic work; 1: <10 h/week; 2: 11–20 h/week; 3: >21 h/week), frequency of physical exercise (1: twice a week or more; 2: once a week; 3: occasionally or never) and smoking habits (0: have never smoked; 1: ex-smoker of at least 6 months standing; 2: smoker, but not daily; 3: daily smoker).

**Fig. 1:**
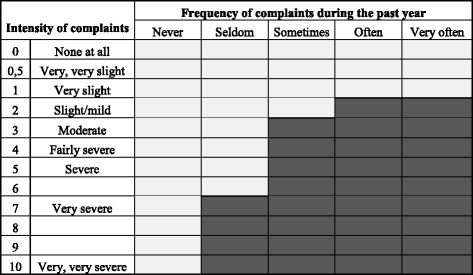
Definition of musculoskeletal *pain*. Definition of considerable musculoskeletal pain, based on the combination of the frequency and the intensity of reported complaints during the preceding year. A subject was considered to have pain (*denoted by dark grey boxes*) if reporting complaints at least “seldom” with an intensity of at least seven (*very severe*), or “sometimes” with an intensity of at least three (*moderate*), or “often” or “very often” with an intensity of at least two (*slight/mild*)

Musculoskeletal pain: the subjects were asked about subjective musculoskeletal complaints in the neck, shoulders, hands, lower back, and feet during the preceding 12 months and 7 days, following the Nordic Questionnaire [19]. In addition, for each body region, information was collected about the frequency of complaints during the past year using a 5-point scale (never, seldom, sometimes, often, or very often [20]) as well as the intensity of complaints on a ten-point-scale, from 0 (none at all) to ten (very, very severe [21]). In this study, a subject was considered to have considerable musculoskeletal pain (subsequently referred to simply as “pain”) if reporting complaints at least “seldom” with an intensity of at least seven (very severe), or “sometimes” with an intensity of at least three (moderate), or “often” or “very often” with an intensity of at least two (slight/mild, see Fig. 1). The condition was defined separately for each body region.

### Clinical examination

A standardised clinical examination of the neck, shoulders, upper back, elbows, wrists, and hands was made of 485 of the subjects [8, 22]. Symptoms and findings were noted, and diagnoses were set by the examiners according to criteria for 19 predefined diagnoses [8]. The prevalence of one or more diagnosed disorders in the neck, shoulders and wrists/hands was calculated. Since the clinical examination only included subgroups of the participants, the existence of diagnoses was not included in the multivariate statistical models.

### Technical measurements of the physical workload

The physical workload was continuously measured by technical measurements during an ordinary work day in 61 right-handed women, distributed among the groups as 13 Te, 12 AnN, 12 TN, 12 AsN and 12 Sg. These individuals were randomly chosen, irrespective of any pain. They were all experienced in their profession, and their mean values were considered as representative of the physical workload for the total population in each group. The mean duration of the recordings was 311 min (range: 190–421 min). Data recorded during lunch and coffee breaks were not included in the analyses.

Inclinometry, based on triaxial accelerometers, was used to measure posture (with angles relative to the line of gravity) and movements for the head, upper back, and both upper arms [23]. The inclinometers were placed with a double-sided adhesive tape on the forehead, to the right of the spine at the level of C7-Th1 (upper back), and on the upper arms. Reference positions were recorded to define the zero for the degree of inclination. All postures during the recordings were calculated in relation to the reference positions, with a sampling frequency of 20 Hz.

Wrist positions and movements were recorded bilaterally using biaxial flexible goniometers [24]. Flexion and extension angles, along with deviation angles, were recorded with a sampling rate of 20 Hz. The angular velocity was used as a measure of movements, and the fraction of time with an angular velocity < 1°/s for continuous time periods of at least 0.5 s, indicated that the hand was held still. The reference position of the wrists (0° of flexion and deviation) was recorded with the arms and hands hanging relaxed alongside the body.

Bipolar surface electromyographic (EMG) registrations were recorded bilaterally for the trapezius muscles and the forearm extensors (m. extensor carpi radialis longus and brevis [25]). Ag/AgCl electrodes were used for the recordings of the muscular activity, at a sampling rate of 1024 Hz. The muscular activity was normalized to the EMG activity (MVE) recorded during maximal voluntary contractions. For EMG, the proportion of time at “rest” was presented (an amplitude < 0.5 % MVE).

For most measures, data were presented as the 50^th^ and/or 90^th^ percentiles of the cumulative distributions during the recording periods. In addition, for upper arm elevation the 99th percentile indicates the peak load. For goniometry, rest (velocity < 1°/s) indicated that the hand was held still. Additional details of the technical measurements are given in the studies of Hansson et al. [23, 24].

To limit the amount of data shown here, we have chosen only to present data for the right hand side shoulder, upper arm, forearm and hand (Additional file 1 Table S1A).

### Statistical analyses

Differences in physical workload among the occupational groups were assessed by questionnaire (MEI and PHYI) and objective technical measurements. As it has been shown that subjects currently suffering from complaints tend to overestimate their physical exposure [26], we also performed sensitivity analyses, and calculated MEI and PHYI for pain-free individuals only. In addition, correlations between self-reported and technically measured physical exposures were calculated using Spearman’s rank correlation coefficient. To assess the potential effects of group affiliation and various exposures to musculoskeletal pain, prevalence ratios (PRs) with 95 % confidence intervals (CIs) were estimated using Poisson regression with unit length of follow up for each study participant. As Te supposedly had the lowest physical work load, they were used as a reference in the group comparisons, which were adjusted for personal and lifestyle factors (age, BMI, number of children at home, personal recovery time, domestic work, physical exercise and smoking). The possible effects of the exposure variables with *p* < 0.30 were initially assessed one-by-one in the regression analysis. Backwards Poisson regression including all exposure measures with *p* < 0.10 was then used to determine the final multivariable models. P-values below 0.05 were regarded as statistically significant when interpreting the results.

## Results

### Self-reported occupational exposures and personal factors

As expected, and intended in the study design, there were differences in the physical workload among the occupational groups. For the self-reported exposure, the mean scores in the MEI (postures and movements) ranged from 16 among the Te to 21 in the TN (Table 1). The AsN reported the highest mean score in the PHYI (physical activity and lifting), while Te and Sg reported the lowest. The sensitivity analyses showed that pain free individuals on average rated MEI one point lower and PHYI 0.4 point lower than individuals with continuing pain (not in the table), but the order among the groups remained. The Te and AnN were less satisfied with the computer workstation arrangements than the other three groups.

The psychosocial measures also indicated a great deal of variation among the groups (Table 1). The Te reported the highest scores compared to the other four groups for job demands and emotional demands. The Te also had the highest perception of job control, while the AsN perceived the lowest. The TN and Sg perceived the highest sensory demands. The mean age, mean BMI and percentage of daily smokers, were higher among the AsN than in the other four groups, while the Sg were, on average, somewhat younger.

### Technical measurements of the physical workload

The self-reported exposures were confirmed by the objective technical measurement of the physical workload (Additional file 1 Table S1A). For example, the recordings indicated that TN worked with a more highly flexed head position than the Sg (head flexion 50th percentile 22° vs. 6.4°, *p* < 0.001). Furthermore, the movement velocities were generally higher among the AsN than in the other groups, (as an example, movement velocities for the right upper arm: 50th percentile AsN 28°/s vs. Sg 12°/s, *p* < 0.001).

### Correlations between self-reported and technically measured physical exposures

The correlations between the self-reported and technically measured physical exposures are given in Additional file 2 Table S2A. Generally, the correlations were highest between self-reported lifting of burdens and physical activity on the one hand, and measured movement velocities on the other. An example would be PHYI and movement velocity in the right arm; 50th percentile; r_s_ = 0.48 (*p* < 0.001). Concerning mechanical exposures, the correlation between the question “Does your job involve working with your arms elevated?” and measured upper arm elevation (90th percentile) was r_s_ = 0.30 (*p* = 0.03) and the correlation between “Does your job involve working with your back bent forwards??” and upper back inclination (90th percentile) was r_s_ = 0.26 (*p* = 0.07).

### Musculoskeletal pain and diagnoses

The prevalence of neck pain was similar (44 %) among Te, AsN and Sg, and lower in AnN (38 %) and TN (35 %, Table 2). The patterns changed for diagnosed disorders in the neck: AnN showed the highest prevalence, while the prevalence in Te and TN was substantially lower. The most common neck diagnoses were tension neck syndrome (12 % of the AnN and 9 % of the AsN; not in the table) and cervicalgia (9 % among the Sg).

**Table 2:** Musculoskeletal pain

Body region	Musculoskeletal pain^a^				Complaints	Diagnosed disorder	
Profession	N	%	Crude PR (CI)	Adjusted^b^ PR (CI)	past 7 days (%)	N	%
Neck							
Te	372	44	1	1	33	96	7.3
AnN	297	35	0.79 (0.65–0.96)	0.77 (0.64–0.94)	27	94	17
TN	305	38	0.86 (0.72–1.03)	0.81 (0.67–0.97)	27	99	7.1
AsN	321	44	1.00 (0.84–1.18)	0.94 (0.79–1.12)	33	93	14
Sg	289	44	1.00 (0.84–1.19)	0.97 (0.82–1.16)	37	103	14
Shoulders							
Te	371	38	1	1	31	96	10
AnN	296	43	1.14 (0.94–1.37)	1.10 (0.91–1.33)	30	94	12
TN	304	44	1.17 (0.97–1.40)	1.11 (0.92–1.33)	32	99	14
AsN	322	49	1.31 (1.10–1.56)	1.22 (1.02–1.45)	35	93	20
Sg	290	51	1.37 (1.15–1.63)	1.32 (1.10–1.57)	43	103	16
Hands							
Te	374	17	1	1	15	96	3.1
AnN	297	21	1.26 (0.92–1.73)	1.24 (0.90–1.71)	17	94	4.3
TN	304	24	1.45 (1.07–1.96)	1.45 (1.07–1.96)	21	99	3.0
AsN	321	37	2.24 (1.71–2.92)	1.94 (1.48–2.56)	33	93	13
Sg	290	25	1.50 (1.11–2.03)	1.61 (1.19–2.18)	24	103	5.8
Lower back						N.a.	
Te	370	36	1	1	20		
AnN	296	38	1.08 (0.89–1.32)	1.06 (0.87–1.30)	26		
TN	304	40	1.11 (0.91–1.34)	1.08 (0.88–1.31)	23		
AsN	320	51	1.42 (1.19–1.69)	1.34 (1.12–1.61)	33		
Sg	290	29	0.82 (0.66–1.03)	0.82 (0.65–1.03)	24		
Feet						N.a.	
Te	375	12	1	1	9		
AnN	296	16	1.32 (0.91–1.92)	1.53 (1.05–2.22)	14		
TN	305	18	1.44 (1.00–2.08)	1.46 (1.02–2.10)	17		
AsN	322	26	2.15 (1.55–2.98)	1.83 (1.31–2.57)	23		
Sg	291	10	0.78 (0.50–1.22)	0.89 (0.57–1.40)	10		

Prevalence of musculoskeletal pain^a^ during the preceding 12 months, in the neck, shoulders, hands, lower back, and feet, among women teachers (Te), anaesthetic nurses (AnN), theatre nurses (TN), assistant nurses (AsN) and sonographers (Sg). Prevalence ratios (PR) and 95 % confidence intervals (CI) are calculated using Poisson regression, both crude, and adjusted for personal and lifestyle factors^b^. The prevalence of complaints in the preceding 7 days are also shown. Among those who participated in the clinical examinations of the neck, shoulders and hands, the prevalence of one or more diagnosed disorders is given.^a^Based on frequency and intensity of musculoskeletal complaints during the past 12 months

^b^Age, BMI, number of children at home, personal recovery time, domestic work, physical exercise and smoking

In the shoulders, the prevalence of pain ranged from 51 % among the Sg to 38 % among the Te (Table 2). Diagnosed disorders in the shoulders were most common in the AsN, while the other groups showed a lower prevalence. The most common shoulder diagnoses were acromioclavicular syndrome in the right side (AsN 12 %, AnN 5 %, TN 8 %, Te 7 % and SG 8 %; not in the table) and supraspinatus tendinitis in the right side (AsN 6 % and TN 5 %).

The AsN were those who were most affected in the wrists and hands. Their prevalence of pain implied more than double the prevalence ratio compared to the Te (Table 2). Thirteen per cent of the AsN had one or more diagnosed hand disorders, of whom 6 % were diagnosed with carpal tunnel syndrome (not in the table). Also, the Sg had a somewhat elevated prevalence of pain and diagnosed disorders in the hands. The prevalence of pain in the lower back and feet were highest among the surgical staff (in particular AsN) and lowest among the Sg. Adjustment of the associations with pain for personal and lifestyle factors did not change the patterns among the occupational groups to any major extent, for any of the body regions (Table 2).

### Bivariate associations with pain

In all body regions, pain was statistically significantly associated with several of the self-reported physical and psychosocial exposures, as well as with personal and lifestyle factors (Table 3). For example, a high MEI was associated with pain in all body regions, and so were high job demands. A high PHYI and low job control was associated with pain in the shoulders, hands, lower back and feet, while high emotional demands were associated with neck pain only.

**Table 3:** Bivariate associations

		Neck (*N* = 1584)		Shoulders (*N* = 1583)		Hands (*N* = 1586)		Lower back (*N* = 1580)		Feet (*N* = 1589)	
	Scale	*p*	PR (CI)	*p*	PR (CI)	*p*	PR (CI)	*p*	PR (CI)	*p*	PR (CI)
Physical workload											
MEI	11–33		**1.05 (1.04**–**1.07)**		**1.06 (1.05**–**1.08)**		**1.07 (1.04**–**1.09)**		**1.05 (1.03**–**1.06)**		**1.06 (1.03**–**1.09)**
PHYI	7–21		1.01 (0.99–1.03)		**1.04 (1.02**–**1.05)**		**1.07 (1.04**–**1.10)**		**1.05 (1.03**–**1.07)**		**1.11 (1.08**–**1.15)**
Computer workstation arrangements		<0.001		<0.001		0.10		0.01		0.07	
Satisfied			1		1		1		1		1
Neutral			1.14 (0.98–1.33)		1.15 (1.00–1.32)		0.96 (0.77–1.20)		1.12 (0.96–1.30)		**1.37 (1.05**–**1.79)**
Dissatisfied			**1.45 (1.25**–**1.67)**		**1.34 (1.16**–**1.53)**		1.21 (0.98–1.50)		**1.26 (1.08**–**1.47)**		1.20 (0.89–1.62)
Psychosocial factors											
Job demands	1–4		**1.51 (1.32**–**1.73)**		**1.46 (1.29**–**1.66)**		**1.47 (1.21**–**1.78)**		**1.55 (1.35**–**1.78)**		**1.68 (1.31**–**2.15)**
Job control	1–4		0.87 (0.74–1.03)		**0.82 (0.71**–**0.95)**		**0.48 (0.38**–**0.61)**		**0.81 (0.69**–**0.95)**		**0.66 (0.49**–**0.89)**
Job support	1–4		0.87 (0.75–1.00)		0.89 (0.78–1.02)		0.85 (0.68–1.06)		**0.80 (0.69**–**0.94)**		0.77 (0.58–1.01)
Emotional demands	0–4		**1.15 (1.07**–**1.25)**		1.05 (0.98–1.13)		1.01 (0.90–1.13)		1.08 (1.00–1.17)		1.12 (0.96–1.31)
Demands of hiding emotions	0–4		**1.12 (1.03**–**1.21)**		**1.09 (1.02**–**1.18)**		1.10 (0.98–1.23)		1.06 (0.97–1.15)		1.06 (0.91–1.23)
Sensory demands	0–4		**1.11 (1.01**–**1.22)**		**1.24 (1.13**–**1.35)**		**1.37 (1.19**–**1.58)**		1.08 (0.98–1.19)		**1.24 (1.04**–**1.48)**
Leadership	0–4		**0.89 (0.82**–**0.96)**		**0.91 (0.84**–**0.98)**		0.93 (0.83–1.05)		**0.86 (0.79**–**0.93)**		0.87 (0.75–1.01)
Self-efficacy	1–5		1.07 (0.94–1.22)		1.08 (0.96–1.22)		**1.22 (1.01**–**1.48)**		1.11 (0.97–1.27)		1.08 (0.84–1.40)
Personal and lifestyle factors											
Age	years		**0.99 (0.99**–**1.00)**		**0.99 (0.99**–**1.00)**		**1.03 (1.02**–**1.03)**		1.00 (1.00–1.01)		**1.03 (1.02**–**1.05)**
BMI	points		1.01 (0.99–1.02)		1.01 (0.99–1.02)		**1.03 (1.01**–**1.05)**		**1.02 (1.00**–**1.04)**		**1.09 (1.07**–**1.11)**
Number of children			1.00 (0.95–1.06)		0.98 (0.93–1.03)		**0.83 (0.76**–**0.91)**		0.96 (0.90–1.02)		**0.76 (0.67**–**0.86)**
Personal recovery time		0.003		0.001		0.93		0.004		0.39	
≥ 3 h /day			1		1		1		1		1
1–2 h/day			1.09 (0.94–1.27)		**1.22 (1.05**–**1.41)**		0.99 (0.80–1.22)		**1.21 (1.03**–**1.43)**		1.09 (0.83–1.42)
< 1 h/day			**1.32 (1.11**–**1.56)**		**1.37 (1.16**–**1.62)**		0.96 (0.74–1.23)		**1.37 (1.14**–**1.65)**		0.88 (0.63–1.24)
Domestic work		0.01		0.04		0.58		0.11		0.90	
0–10 h/week			1		1		1		1		1
11–20 h/week			**1.17 (1.02**–**1.35)**		1.07 (0.94–1.23)		0.91 (0.75–1.11)		1.15 (0.99–1.33)		0.95 (0.74–1.23)
≥ 21 h/week			**1.26 (1.07**–**1.47)**		**1.20 (1.04**–**1.39)**		0.91 (0.72–1.15)		1.16 (0.99–1.38)		0.94 (0.70–1.26)
Physical exercise		0.33		0.84		0.18		0.28		0.52	
Twice a week or more			1		1		1		1		1
Once a week			1.04 (0.88–1.24)		0.97 (0.82–1.14)		0.77 (0.58–1.02)		1.03 (0.86–1.24)		0.99 (0.71–1.38)
Occasionally or never			1.13 (0.96–1.34)		1.03 (0.88–1.21)		1.01 (0.78–1.30)		1.15 (0.97–1.37)		1.19 (0.87–1.63)
Smoking habits		0.09		0.03		0.001		0.04		0.004	
Have never smoked			1		1		1		1		1
Former smoker			1.07 (0.94–1.22)		1.03 (0.91–1.17)		**1.28 (1.07**–**1.55)**		1.11 (0.97–1.28)		**1.47 (1.16**–**1.87)**
Smoking, but not daily			1.28 (0.98–1.66)		0.98 (0.73–1.32)		0.82 (0.47–1.41)		1.26 (0.95–1.67)		0.92 (0.47 (1.79)
Daily smoker			1.26 (0.99–1.60)		**1.36 (1.12**–**1.66)**		**1.73 (1.27**–**2.35)**		**1.35 (1.06**–**1.72)**		**1.66 (1.08**–**2.56)**

Bivariate associations in the total study population, between musculoskeletal pain during the preceding 12 months in the neck, shoulders, hands, lower back, and feet, and self-reported physical workload, psychosocial, personal and lifestyle factors. These were calculated using Poisson regression and the prevalence ratio (PR) and 95 % confidence intervals (CI) are shown. Overall *p*-values are given for categorical variables. Results in bold face are statistically significant

### Final multivariable models

Among physical factors, high MEI was strongly associated with an increased risk of pain in the neck, shoulders, hands and lower back, while a high PHYI was associated with pain in the feet (Table 4). Dissatisfaction with the computer work station arrangement was associated with increased risk of pain in the neck and the shoulders. Concerning psychosocial factors, perceived high job demands were associated with pain in all body regions. Young age was associated with an increased risk of neck pain, while an older age was associated with an increased risk of pain in the hands and feet. A high BMI was associated with pain in the feet. Among the lifestyle factors, lack of time for personal recovery was associated with increased risk of pain in the shoulders and lower back. Job support, leadership, self-efficacy, domestic work, physical exercise and smoking habits did not qualify for the final model in any body region.

**Table 4:** Final multivariable model

		Neck (*N* = 1424)		Shoulders (*N* = 1419)		Hands (*N* = 1431)		Lower back (*N* = 1406)		Feet (*N* = 1440)	
	Scale	*p*	PR (CI 95 %)	p	PR (CI 95 %)	*p*	PR (CI 95 %)	*p*	PR (CI 95 %)	*p*	PR (CI 95 %)
Physical workload											
MEI	11–33	<0.001	1.07 (1.05–1.10)	<0.001	1.05 (1.03–1.06)	0.001	1.04 (1.02–1.07)	<0.001	1.03 (1.02–1.05)		
PHYI	7–21	<0.001	0.96 (0.93–0.98)							<0.001	1.09 (1.05–1.12)
Computer workstation arrangements		0.03		0.03							
Satisfied			1		1						
Neutral			1.08 (0.93–1.26)		1.15 (0.99–1.32)						
Dissatisfied			1.23 (1.05–1.43)		1.22 (1.05–1.41)						
Psychosocial factors											
Job demands	1–4	0.008	1.26 (1.06–1.49)	0.003	1.24 (1.07–1.43)	0.002	1.40 (1.13–1.74)	<0.001	1.46 (1.24–1.71)	0.03	1.35 (1.03–1.79)
Job control	1–4					<0.001	0.51 (0.40–0.66)	0.04	0.83 (0.70–0.99)	0.05	0.72 (0.52–1.00)
Emotional demands	0–4	0.04	1.10 (1.00–1.20)								
Sensory demands	0–4	0.07	0.90 (0.81–1.01)					0.06	0.89 (0.80–1.00)		
Personal and lifestyle factors											
Age	years	0.05	0.99 (0.99–1.00)			<0.001	1.02 (1.01–1.03)			<0.001	1.03 (1.01–1.04)
BMI	points					0.06	1.02 (1.00–1.04)	0.06	1.02 (1.00–1.03)	<0.001	1.08 (1.06–1.10)
Number of children						0.05	0.90 (0.82–1.00)	0.04	0.93 (0.88–1.00)	0.05	0.87 (0.75–1.00)
Personal recovery time				0.02				0.04			
≥ 3 h /day					1				1		
1–2 h/day					1.24 (1.06–1.45)				1.15 (0.97–1.36)		
< 1 h/day					1.25 (1.05–1.49)				1.29 (1.06–1.58)		

Final multivariable model in the total study population of associations between musculoskeletal pain during the preceding 12 months in the neck, shoulders, hands, lower back and feet, and self-reported physical workload, psychosocial, personal, and lifestyle factors. These were calculated using Poisson regression (backward selection). *P*-values (overall in categorical variables), prevalence ratio (PR) and 95 % confidence intervals (CI) are shown.The variables job support, leadership, self-efficacy, domestic work, training and smoking habits did not qualify for the final model in any body region

An unexpected finding was that a high PHYI, which was positively associated with pain in the bivariate analyses, was protective against neck pain in the multivariable model. There was also a tendency indicating that high sensory demands were protective, in contrast to the findings in the bivariate analyses. In Table 5, a layered cross-tabulation of pain in the total study population across dichotomized values of sensory demands, MEI and PHYI is illustrated. This in-depth analysis indicated that high PHYI was protective only for the combination of high sensory demands and high MEI (prevalence of neck pain decreased from 53 to 44 % when moving from low to high PHYI; Table 5, last row). No marked protective effect of a high PHYI was seen for any other combination of sensory demands and MEI.

**Table 5:** Layered cross-tabulation

Sensory demands			Low PHYI		High PHYI	
		MEI	N	%	N	%
Low sensory demands	Low MEI		350	36	116	34
	High MEI		68	41	143	44
High sensory demands	Low MEI		150	31	91	36
	High MEI		162	53	374	44

The prevalence (%) of neck pain in the total study population across combinations of dichotomized values (high and low) in sensory demands, mechanical exposure index (MEI) and physical exposure index (PHYI)

## Discussion

Neck pain was equally frequent among teachers, assistant nurses and sonographers, while less frequent among anaesthetic and theatre nurses. The sonographers suffered the highest prevalence of shoulder pain, while the assistant nurses were the most affected in the wrists and hands, lower back and feet.

As expected, there were major differences between the occupational groups as to the physical and psychosocial work environment. Both the physical workload and the psychosocial work environment were associated with pain, in all body regions, though different factors affected different regions. Pain in the neck, shoulders, hands and lower back were strongly associated with a high MEI (strenuous work postures and movements) and high job demands, while pain in the feet was associated with a high PHYI (walking, lifting, materials handling) and a high BMI. Young age was associated with pain in the neck, while older age was associated with pain in the hands and feet. Lack of time for personal recovery was associated with pain in the shoulders and lower back.

### Methodological considerations

As in all studies based on self-reported exposure and self-reported health, the results must be interpreted with caution. It is well known that individuals with continuing pain perceive their exposure (physical and psychosocial) to be more demanding than individuals without pain, and they therefore may have overestimated their exposure [26]. However, in sensitivity analysis with pain-free individuals only, the differences among the occupational groups in self-reported occupational factors remained.

Data was collected during an extended period of time. However, we alternated the dispatch of questionnaires between the occupational groups: we began with a surgery department, then a school, then some sonography departments, and then another surgery department, and so on. Therefore, we do not believe that the results were affected to any significant extent by any changes in society that may have occurred during this time period.

Technical measurements of the physical workload, which provide objective data on physical workloads, independent of the examiner, confirmed the differences among the occupational groups. However, because such measurements are time-consuming, we could only study a limited number of individuals in each occupation. The measurements could thus not be included in the multivariable models, and instead we used self-reported physical exposures. We also studied the extent to which the objective technical measurements showed co-variance with the issue of posture and physical strain in the questionnaire. The self-reported exposures referred to longer periods of time, while the technical measurements refer only to the day on which the subjects carried the measuring equipment, which might be either more or less physically demanding than usual. Also, the items in the questionnaire do not entirely correspond with the dimensions we are able to register with technical measurements. Nevertheless, we consider the correlations between the technical measurements and the self-reported physical exposure to be fairly high. Thus, we believe that the self-reported data on physical exposure represent reasonable assessments of the actual exposure.

Musculoskeletal disorders are known to have an intermittent pattern of recurrence [27, 28]. In the same individual, complaints may come and go, and their intensity may vary at over time. Thus, asking about pain during an extended period of time is valuable when compared to reports of only currently-present pain, or pain during the past week. In addition, the severity of complaints varies among individuals: some may have slight or mild complaints that do not interfere with their daily life; others have serious problems which may result in taking long term sick leave or a disability pension. Such variations are not captured by questions which simply address complaints (“any pain”). Therefore, we defined cases of pain based on the subjects’ reports on frequency as well as the intensity of complaints during the preceding 12 months. Such an approach has not, to our knowledge, been used earlier, and we consider this a major strength of the study.

The frequently used Nordic questionnaire [19] with measures of musculoskeletal complaints in the preceding 7 days gave somewhat differing results, when compared to those for pain. In the total study population, 489 subjects (31 %) reported complaints from the neck in the preceding 7 days, 631 subjects (41 %) fulfilled the criteria for neck pain, of which 421 (27 %) were included in both case-definitions. Hence, 4 % of the subjects reported complaints during the preceding 7 days, but these were not so severe that they met the criteria for neck pain. At the same time, the pain-measure captured 210 other women who did not have current complaints, but had been more seriously affected at some point in the preceding 12 months.

### Diagnosed disorders and pain

Regarding diagnosed disorders, the present occupational groups may be compared to reference material covering 1762 women in 20 different occupational groups, examined by the same method of clinical examination [8]. This reference material represents diverse occupations with a diversity of work tasks and varying socioeconomic status. In this context, the occupational groups in this study had low or medium prevalence of one or more diagnosed disorders in the neck and shoulders.

An interesting finding was that the Te reported a relatively high prevalence of neck pain while the prevalence of diagnosed neck disorder was low. The opposite was true among the AnN who had a lower prevalence of neck pain than the teachers, but a considerably higher prevalence of diagnosed neck disorders. Also, the AnN had a higher physical workload than the Te, while the Te reported higher psychosocial scores than the AnN. Thus, the results suggest that the adverse psychosocial conditions among the teachers give rise to a different kind of pain in the neck than that from physical overload; one which is troublesome, but not as severe as that afflicting the nurses [29].

The AsN were the most affected as regards pain in the hands, even after adjustment for their higher mean age and BMI. They also had a high prevalence of diagnosed disorders in the hands, as compared to the occupational groups studied by Nordander et al. [8]. The prevalence of AsN in the present work was at the same level as women in repetitive or constrained work, a majority of whom worked in industrial settings [8].

The prevalence of pain in the feet was significantly higher in the surgical staff (in particular AsN), than in Te and Sg. While the Sg and Te to some extent were seated, the work tasks in the surgical ward were characterized by standing, walking, lifting and carrying. In such exposures, a high BMI amplifies the load on the feet.

### Associations with risk factors

The strong association between a high MEI and musculoskeletal pain was expected. Since lifting and materials handling result in high loads in these body regions, one could rather expect a high PHYI to be one of the most important risk factors. However, in spite of bivariate associations between a high PHYI and pain in the shoulders, hands and lower back, a high PHYI did not qualify for the final multivariable models. On the other hand, a high PHYI was strongly associated with pain in the feet. For subjects with a combination of high MEI and high sensory demands, a high PHYI was protective against pain in the neck. One may speculate that a high PHYI in this subgroup reflects a physically more varied work, which is considered to be favourable [30].

As reported earlier [31], dissatisfaction with the computer workstation arrangements was associated with pain in the neck and shoulders. However, due to the cross-sectional nature of the data, we do not know if individuals with pain reported more complaints on the computer work station arrangements, or if awkward work postures during computer work are the cause of the pain [32].

High job demands were associated with musculoskeletal pain in all body regions. In the job demand dimension of the questionnaire, the necessity for hard work, a high work pace or stressful work were queried; these include both psychosocial and physical exposures. Thus, as in the case of tasks with a high perceived MEI, job demands may be perceived as more onerous by individuals with continuing pain.

Several studies indicate an association between low job control and neck complaints [5, 33], but after adjustments, this was not evident in our study population. However, low job control was strongly associated with pain in the hands. This may be explained by the fact that the occupational groups with the highest prevalence of pain in the hands (AsN and Sg) also performed force-demanding or hand-intensive work tasks. At the same time, AsN and Sg reported the lowest scores in job control. As discussed in the paper by Nordander et al. [8], low job control and repetitive or hand intense work are strongly correlated, and thus could not be distinguished. Therefore, we consider the physical factors, rather than the psychosocial ones, to be the cause of the hand pain.

While the association between high emotional demands and neck pain was expected, we did not expect to find that high sensory demands tended to protect against pain in the neck and lower back. We have no explanation for this, but high sensory demands may be present in stimulating work tasks, such as those among the theatre nurses, and thus are associated with a protective effect.

The prevalence of musculoskeletal disorders increase with age in the general population [34]. Interestingly, and contrary to common belief, the younger women showed a higher prevalence of neck pain than the older ones, even after adjustment for occupational factors in the multivariable model. Similar results have been reported earlier among computer workers [35]. In contrast, pain in hands and feet there were strongly associated with increasing age. These results suggest that there may be different mechanisms of pain in the neck, as compared to pain in the hands and feet.

## Conclusions

Although none of the studied occupations had an extremely high prevalence of pain, both physical and psychosocial factors were associated with pain. However, the occupational groups were affected differently, and need different protective measures. Thus, to prevent pain among teachers, where the physical workload was lower than in the other groups, the focus should be on improving the psychosocial work environment in terms of reduced emotional demands and workload. In contrast, the surgical staff and sonographers may benefit from preventive measures addressing the physical work load, such as lifting and constrained postures, and, in particular, reducing hand force demands among assistant nurses.

## Additional files

Additional file 1: Table S1. A. Technical measurements of the physical workload. (DOCX 18 kb) Additional file 2: Table S2. A. Correlations between self-reported and technically measured physical workload (DOCX 24 kb)

## Abbrevations

AnN: Anaesthetic nurses
AsN: Assistant nurses
BMI: Body mass index
MEI: Mechanical exposure index
PHYI: Physical exposure index
Sg: Sonographers
Te: Teachers
TN: Theatre nurses
