# Supplementary material for: Cross-sectional associations between occupational factors and musculoskeletal pain in women teachers, nurses and sonographers
Source: BMC Musculoskelet Disord. 2016 Jan 18;17:35. doi: 10.1186/s12891-016-0883-4 (PMC4717636; doi:10.1186/s12891-016-0883-4)
Supplement: Additional file 1: Table S1. — A. Technical measurements of the physical workload. (DOCX 18 kb) [file 12891_2016_883_MOESM1_ESM.docx]

**Additional file 1: Table S1. Technical measurements of the physical workload**. Technically measured physical workload (means, with standard deviations within parentheses) in the head, upper back and right shoulder, upper arm, forearm and hand, among teachers (Te), anaesthetic nurses (AnN), theatre nurses (TN), assistant nurses (AsN) and sonographers (Sg); all females.

|  | Percentile  distribution | Te  (n=13) | AnN  (n=12) | TN  (n=12) | AsN  (n=12) | Sg  (n=12) |
| --- | --- | --- | --- | --- | --- | --- |
|  |  |  |  |  |  |  |
| Neck/upper back/shoulder/arm |  |  |  |  |  |  |
| Postures and movements |  |  |  |  |  |  |
| Head |  |  |  |  |  |  |
| Posture (°)* | 50^th^ | 14 (6.1) | 15 (5.8) | 22 (7.0) | 12 (5.6) | 6.4 (4.8) |
|  | 90^th^ | 43 (9.2) | 45 (6.3) | 42 (4.2) | 42 (6.8) | 37 (5.0) |
| Velocity (°/s) | 50^th^ | 9.0 (2.1) | 8.4 (2.2) | 9.1 (1.6) | 13 (3.0) | 6.8 (2.0) |
| Upper back |  |  |  |  |  |  |
| Posture (°)* | 50^th^ | 15 (4.1) | 11 (5.5) | 12 (4.5) | 10 (4.4) | 10 (4.4) |
|  | 90^th^ | 35 (4.8) | 30 (8.5) | 27 (5.7) | 28 (4.9) | 25 (7.0) |
| Velocity (°/s) | 50^th^ | 5.8 (1.7) | 5.7 (2.1) | 6.4 (1.1) | 9.0 (1.7) | 4.1 (1.3) |
| Upper arm |  |  |  |  |  |  |
| Elevation (°) | 50^th^ | 27 (3.2) | 28 (3.8) | 29 (4.6) | 25 (3.9) | 30 (4.0) |
|  | 99^th^ | 88 (12) | 89 (14) | 93 (12) | 95 (14) | 73 (6.7) |
| Velocity (°/s) | 50^th^ | 17 (5.7) | 17 (6.9) | 21 (4.0) | 28 (5.4) | 12 (3.8) |
| Muscular load |  |  |  |  |  |  |
| Trapezius |  |  |  |  |  |  |
| Rest (% time) | Na | 17 (9.8) | 13 (7.8) | 18 (14) | 9.7 (9.4) | 22 (13) |
| Activity (% max MVE) | 90^th^ | 13 (4.1) | 12 (3.2) | 12 (4.4) | 15 (3.7) | 11 (4.0) |
|  |  |  |  |  |  |  |
| Forearm/hand |  |  |  |  |  |  |
| Postures and movements |  |  |  |  |  |  |
| Wrist** |  |  |  |  |  |  |
| Posture (°)*** | 50^th^ | -12 (5.7) | -11 (5.5) | -10 (5.2) | -8.7 (7.4) | -20 (10) |
| Movements |  |  |  |  |  |  |
| Rest < 1°/s (% of time) | Na | 5.1 (2.2) | 6.2 (4.0) | 4.9 (1.9) | 4.2 (2.5) | 5.7 (3.9) |
| Velocity (°/s) | 50^th^ | 6.4 (3.2) | 6.1 (3.0) | 6.9 (3.0) | 9.7 (3.9) | 5.0 (2.1) |
| Muscular load |  |  |  |  |  |  |
| Forearm extensors |  |  |  |  |  |  |
| Rest (% time) | Na | 8.4 (6.7) | 13 (5.3) | 10 (8.5) | 13 (5.5) | 7.1 (4.7) |
| Activity (% max MVE) | 90^th^ | 17 (9.1) | 20 (8.0) | 22 (13) | 20 (7.6) | 18 (5.3) |
|  |  |  |  |  |  |  |

* Positive values indicate forward flexion

** One theatre nurse missing due to technical error

*** Positive values indicate palmar flexion
