# Supplementary material for: Cross-sectional associations between occupational factors and musculoskeletal pain in women teachers, nurses and sonographers
Source: BMC Musculoskelet Disord. 2016 Jan 18;17:35. doi: 10.1186/s12891-016-0883-4 (PMC4717636; doi:10.1186/s12891-016-0883-4)
Supplement: Additional file 2: Table S2. — A. Correlations between self-reported and technically measured physical workload (DOCX 24 kb) [file 12891_2016_883_MOESM2_ESM.docx]

**Additional file 1: Table S2**. **Correlations between self-reported and technically measured physical workload.** Correlation matrix (Spearman rank-correlation coefficients; r_s_) for the group means of technically measured physical exposures (postures, movement velocities and muscular load) and reported workload in relevant items of the mechanical and physical exposure indices, among subjects who participated in both examinations (n=51-53). The correlations are illustrated in different shades of white and grey; from lowest (white squares R=0.0 – 0.15) to highest (dark grey squares R=0.45-0.60). Positive (+) and negative correlations (-) are indicated. The corresponding p-values are below 0.05 for r_s_ =0.28, below 0.01 for r_s_ =0.36 and below 0.001 for r_s_ =0.45.
